# Limited polymorphism in k13 gene of *Plasmodium falciparum* and k12 of *Plasmodium vivax* isolates imported from African and Asian countries between 2014 and 2019 in Hangzhou city, China

**DOI:** 10.1186/s12879-021-06579-6

**Published:** 2021-08-21

**Authors:** Xingyi Jin, Sujuan Zhu, Weimin Xu, Junfang Chen, Wei Ruan, Xiaoxiao Wang

**Affiliations:** 1 Hangzhou Center for Disease Control and Prevention, Hangzhou, 310021 China; 2 Zhejiang Provincial Center for Disease Control and Prevention, Hangzhou, 310051 China

**Keywords:** Polymorphism, *P. falciparum*, *P. vivax*, k13

## Abstract

**Background:**

Malaria causes major public health problems globally and drug resistance hinders its control and elimination. Molecular markers associated with drug resistance are considered as a beneficial tool to monitor the disease trends, evolution and distribution so as to help improve drug policy.

**Methods:**

We collected 148 *Plasmodium falciparum* and 20 *Plasmodium vivax* isolates imported into Hangzhou city, China between 2014 and 2019. k13 gene of *P. falciparum* and k12 of *P. vivax* were sequenced. Polymorphisms and prevalence of k13 and k12 were analyzed.

**Results:**

Most (98.65%, 146/148) *P. falciparum* infections were imported from Africa, and half *P. vivax* cases came from Africa and the other half from Asia. Nucleotide mutation prevalence was 2.03% (3/148) and the proportion of amino acid mutations was 0.68% (1/148). The amino acid mutation, A676S, was observed in an isolate from Nigeria. No mutation of k12 was observed from the parasites from African and Asian countries.

**Conclusions:**

Limited polymorphism in k13 gene of *P. falciparum* isolates imported from African countries, but no evidence for the polymorphism of k12 in *P. vivax* samples from African and Asian countries was found. These results provide information for drug policy update in study region.

**Supplementary Information:**

The online version contains supplementary material available at 10.1186/s12879-021-06579-6.

## Introduction

Malaria is one of the most important public health problems worldwide. According to the 2020 World Malaria Report, there were an estimated 229 million cases in 2019. Of the five malaria species, *Plasmodium falciparum* caused the largest number of cases worldwide, and *Plasmodium vivax* was the most widely distributed [1].

Since the use of antimalarial drugs began in the 1940s, drugs such as chloroquine, mefloquine, quinine, pyrimethamine and sulfadoxine have played an important role in malaria prevention and control. However, with the long-term and widespread use of antimalarial drugs, drug resistance has developed [2, 3]. To date, *P. falciparum* has developed varying degrees of resistance to all commonly used antimalarial drugs, such as chloroquine, artemisinin and sulfadoxine–pyrimethamine [4–6]. *P. vivax* has also shown resistance to antimalarial drugs [7]. Artemisinin-based combination therapy (ACT) has been suggested as the first-line treatment by the World Health Organization (WHO) since 2006, and its widespread use has led to concerns about artemisinin resistance being raised by researchers and local health authorities. The emergence and development of antimalarial drug resistance not only threatens malaria patients, but also seriously hinders the process of malaria elimination. Therefore, it is important to monitor antimalarial drug resistance, thereby gaining experience on which to base further improvements of treatment protocols.

There are four methods used to monitor drug resistance [8]. Compared to therapeutic efficacy studies, in vitro tests, and measurement of drug concentration, use of molecular markers has the following advantages: resistance-related mutations easily obtained by molecular experiments, avoiding host confounding factors, and blood filter paper samples are more easily transported and stored [8]. Furthermore, multiple samples can be detected in a batch. So, it was preferred as a tool to monitor drug resistance. For *P. falciparum*, Kelch 13-propeller(k13) mutations have been identified as molecular makers of partial artemisinin resistance [1]. To date, 10 markers have been validated as being associated with resistance to artemisinin: F446I, N458Y, M476I, Y493H, R539T, I543T, P553L, R561H, P574L and C580Y, whereas 11 markers are considered as candidates [1, 9–12]. Regarding *P. vivax*, it is assumed that the widespread use of ACT also exerts similar selective pressure on *P. vivax* parasites [13]*.* k12, k13 orthologue in *P. vivax* (here named k12 gene because of its location on chromosome 12), has been investigated by recent studies. Studies from Cambodia and the China–Myanmar border have found k12 mutations (V552I in Cambodia and M124I on China–Myanmar border) [13, 14]. However, another study from Cambodia by longitudinal pooled deep sequencing of k12 gene suggested a lack of selection by artemisinin [15]. Therefore, more evidence is required to clarify its polymorphism in parasite population as well as its potential association with artemisinin resistance.

Hangzhou city, located in the east of China. About thirty malaria cases were imported to Hangzhou each year from Africa and other countries of Asia. To reduce the risk of reemergence due to malaria importation, a highly effective surveillance system was implemented to find each imported case and stop malaria spreading, following national “1-3-7” strategy. And each infection was laboratory-confirmed and epidemiologically investigated. In this study, *P. falciparum* and P. vivax samples were obtained from imported malaria cases in Hangzhou from 2014 to 2019. k13 gene of *P. falciparum* and k12 of *P. vivax* were sequenced to identify and track the prevalence of drug resistance in the countries of origin, so as to provide evidence for refinement of drug policy.

## Materials and methods

### Study site

Hangzhou city (longitude 118°20′–120°37′, latitude 29°11′–30°34′) is located in the eastern Zhejiang province, China. The dominant mosquito species in Hangzhou city has been *Anopheles sinensis* over the past 40 years. Since 2010, all the malaria cases in Hangzhou were imported, most of which returned back from Africa, followed by southeast Asia.

### Sample collection and DNA extraction

We investigated 148 cases of *P. falciparum* and 20 of *P. vivax* malaria that were imported into Hangzhou city between 2014 and 2019. According to the national guideline on malaria diagnosis, individuals with malaria-related symptoms, and positive by microscopy or rapid diagnostic tests (RDTs) would be reported by hospitals or clinics in Hangzhou. Local Center for Disease Control and Prevention carried out epidemiological investigation for each patient, including detailed travelling history to track parasite origins. Infections were double-checked for species by PCR in Zhejiang Provincial Center for Disease Control and Prevention. According to national regulation of antimalarial drugs, for uncomplicated *P. falciparum* infections, Dihydroartemisinin and Piperaquine phosphate was the first-choice. And for *P. vivax* patients, Chloroquine Phosphate was the first line drug, followed by Dihydroartemisinin and Piperaquine phosphate. Approximately 1 ml of venous blood was obtained from each patient before treatment. All the blood samples were stored at − 80 °C until use. Genomic DNA was extracted by QIAamp DNA Mini kit (Qiagen Inc., Hilden, Germany).

### DNA amplification and sequencing

The propeller region of k13 gene of *P. falciparum* isolates was amplified by nested PCR as previously described [16], whereas k12 gene of *P. vivax* isolates was amplified by regular PCR using proof-reading polymerase (see Additional file 1: Table S1). Because of its long sequence, k12 gene was divided into two segments for amplification. Amplification products were then sequenced by Sangon Biotech Co. Ltd. (Shanghai, China). Oligonucleotide primers and cycling conditions are listed in Additional file 1: Table S1). A final 25-μl reaction volume was used.

### Data analysis

Nucleotide and amino acid sequences of k13 gene were aligned and compared with reference sequences from NCBI database by Mega version7.0.26 (https://www.megasoftware.net/). The k13 sequence of PF3D7_1343700(MF285413.1) and k12 sequence of Salvador I (Sal-I) genome (PVX_083080) were used as references. A database was constructed using Microsoft Excel 2017. The mutant and wild-type alleles of the collected samples were used to generate the prevalence of the alleles.

## Results

### General information

**Fig. 1:**
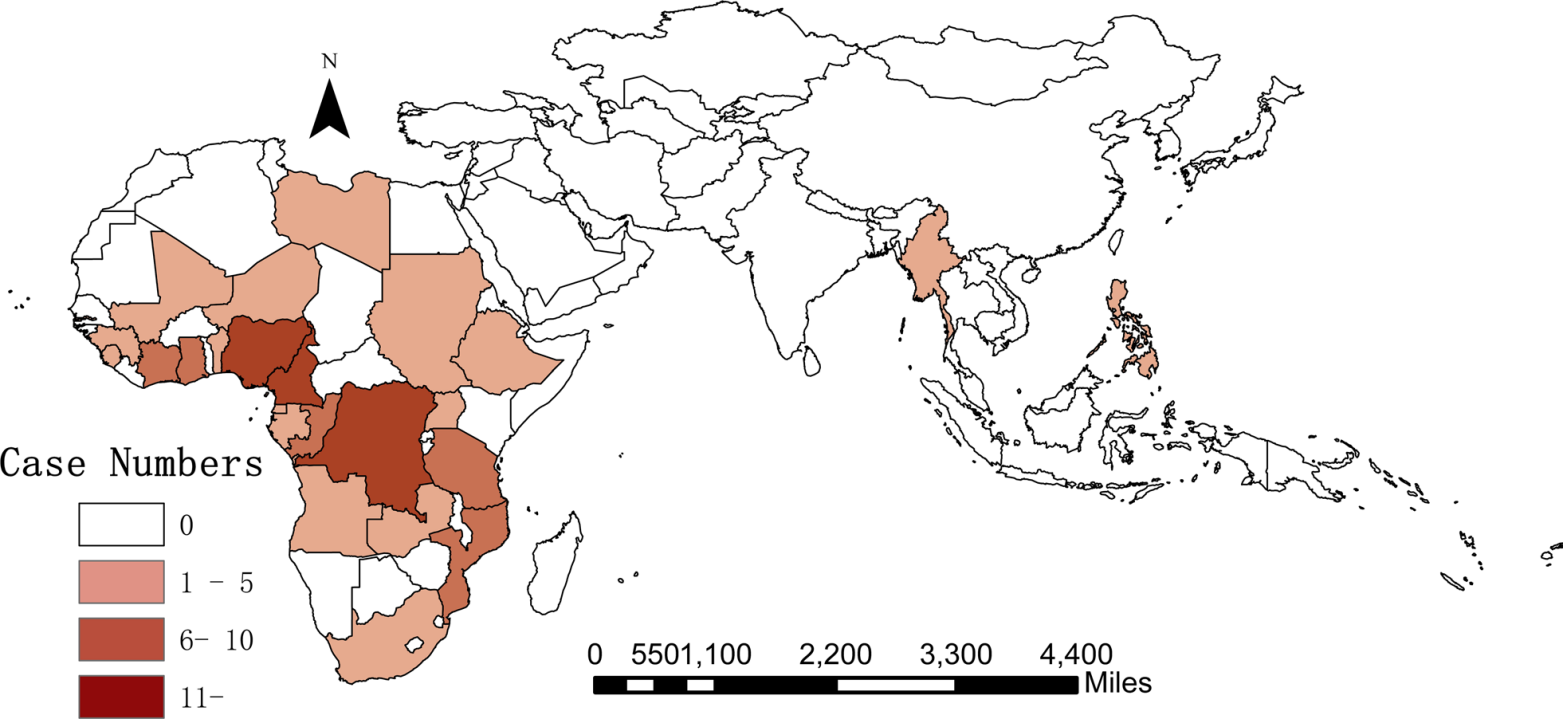
Geographical distribution of imported *P. falciparum* cases from Africa and Asia

**Fig. 2:**
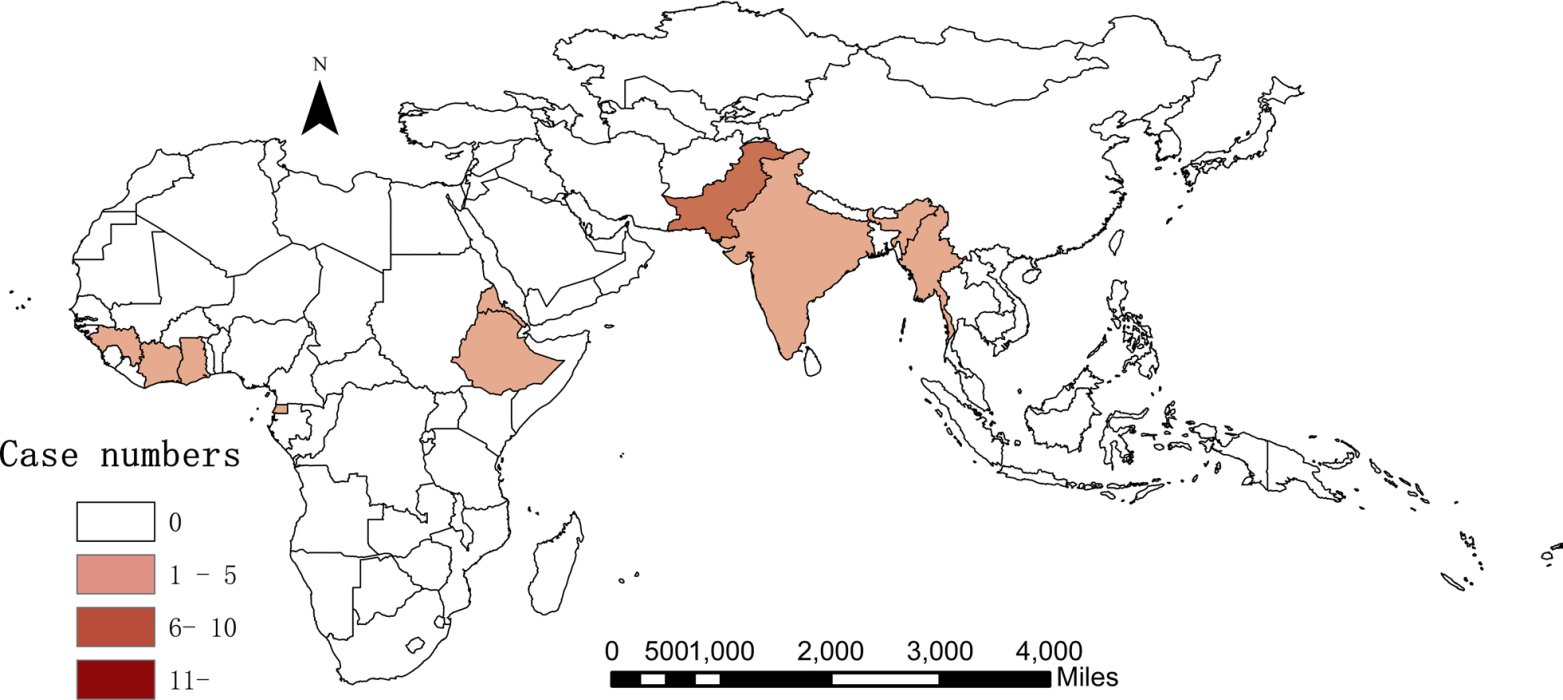
Geographical distribution of imported *P. vivax* cases from Africa and Asia

We collected 168 samples of malaria infections imported from Africa and other countries of Asia to Hangzhou city, China, including 148 *P. falciparum* and 20 *P. vivax* cases (Tables 1 and 2). Specifically, 98.65% (146/148) *P. falciparum* infections were imported from Africa, while two cases returned back from Asia. Nigeria (31.08%, 46/148), Cameroon (11.49%,17/148), Democratic Republic of the Congo (8.11%, 12/148) and Ghana (6.76%, 10/148) were the main importing countries (Fig. 1). For imported cases of vivax malaria, half came from Africa and half from Asia. Most *P. vivax* infections (30%,6/20) were imported from Pakistan, followed by Ethiopia (25%, 5/20) (Fig. 2).

**Table 1:** Distribution of imported *P. falciparum* cases of Hangzhou city between 2014 and 2019

Region	Country	Year						Total no. of cases
		2014	2015	2016	2017	2018	2019	
*Central Africa*		*6*	*8*	*9*	*6*	*8*	*4*	*41*
	Cameroon	1	3	2	3	6	2	17
	Congo	3	2	1	1			7
	DR.Congo		2	6	2	1	1	12
	Equatorial Guinea	1					1	2
	Gabon	1	1			1		3
*East Africa*		*1*	*1*	*2*		*4*	*2*	*10*
	Ethiopia					1		1
	Tanzania	1	1	1		2	2	7
	Uganda			1		1		2
*North Africa*		*2*		*1*				*3*
	Sudan	2		1				3
*South Africa*		*2*	*1*	*8*	*3*		*2*	*16*
	Mozambique		1	3	2			6
	Angola	2		1			2	5
	Republic of South Africa			2	1			3
	Zambia			2				2
*West Africa*		*14*	*6*	*17*	*10*	*18*	*11*	*76*
	Republic of Niger				1			1
	Ghana	1		1	3	4	1	10
	Benin	2		1		1		4
	Côte d'Ivoire	1	1			1	3	6
	Guinea					1		3
	Liberia						3	3
	Mali			1				1
	Nigeria	10	4	12	6	10	4	46
	Republic of The Gambia		1					1
	Sierra Leone					1		1
*Asia*				*1*	*1*			*2*
	Myanmar				1			1
	Pilipinas			1				1
Total		25	16	38	20	30	19	148

**Table 2:** Distribution of imported *P. vivax* cases of Hangzhou city between 2014 and 2019

Region	Country	Year						Total no. of cases
		2014	2015	2016	2017	2018	2019	
*Asia*		*1*	*3*	*3*	*2*	*0*	*1*	*10*
	Myanmar		2					2
	India	1			1			2
	Pakistan		1	3	1		1	6
*East Africa*		*0*	*2*	*0*	*3*	*1*	*0*	*6*
	Ethiopia		2		3			5
	Eritrea					1		1
								2
*West Africa*		*0*	*1*	*1*	*0*	*1*	*0*	*3*
	Côte d'Ivoire					1		1
	Ghana			1				1
	Guinea		1					1
*Central Africa*		*0*	*1*	*0*	*0*	*0*	*0*	*1*
	Equatorial Guinea		1					1
Total		1	7	4	5	2	1	20

### k13 propeller polymorphisms

The sequences of 148 k13 nested PCR products were successfully obtained from 148 isolates. The k13 SNPs were analyzed by comparing with the reference. Sequence alignment revealed that three samples harbored a nucleotide mutation, G to T at position 2026, in one isolate from Nigeria, C to G at position 1767 in one isolate from Nigeria, and C to T at position 1479 in one isolate from Côte d'Ivoire. The nucleotide mutation prevalence was 2.03% (3/148). Nucleotide mutation at position 2026 resulted in non-synonymous mutation of amino acid A676S, while nucleotide mutations at positions 1767 and 1479 did not bring in non-synonymous amino acid mutations. The prevalence of amino acid mutation was 0.68% (1/148) (Table 3).

**Table 3:** Prevalence of mutations in the k13 propeller domain of imported *P. falciparum* infections of Hangzhou city between 2014 and 2019

Point mutations		Location and year
Nucleotide	Amino acid	
G2026T	A676S	Nigeria, 2016
C1767G	V589V	Nigeria, 2016
C1479T	Y493Y	Côte d'Ivoire, 2018

### k12 propeller polymorphisms

k12 of all *P. vivax* isolates was successfully amplified and sequenced, and compared with the reference. All the k12 of 20 imported *P. vivax* cases were identical to the reference sequence. Namely, no mutation of k12 was observed from the isolates from Africa and Asia.

## Discussion

The current study aimed at providing surveillance information for artemisinin resistance by analyzing the k13 propeller domain in *P. falciparum* and k12 in *P. vivax* isolates imported from African and Asian countries between 2014 and 2019 in Hangzhou city, China. Our results showed that both nucleotide and amino acid mutations in *P. falciparum* isolates were at very low frequencies. As for *P. vivax*, we demonstrated that k12 in *P. vivax* isolates was highly conserved.

Mutations in the k13 propeller domain are important determinants of artemisinin resistance [9]. Therefore, k13 marker studies help to identify and track the prevalence of molecular mutations associated with artemisinin partial resistance, as WHO has established a validated list of mutation markers. Since the first discovery of artemisinin resistance in western Cambodia and the border between Cambodia and Thailand in 2008 [5, 6], k13 mutations have become widespread across southeast Asia [17]. In Africa, even slow-clearing infections after ACT treatment were observed at a low frequency of 1%. A578S was the most frequently reported mutation of k13 propeller domain in Africa, and it did not confer artemisinin resistance in vivo or in vitro [18], so the situation concerning artemisinin resistance was thought to be optimistic [19]. However, a recent study reported that an indigenous k13 R561H mutation was identified in 19 samples between 2013 and 2015 from Rwanda and gene editing confirmed that this mutation can drive artemisinin resistance in vitro [10]. It raised international concern for the epidemiology of the k13 marker in Africa. So, the survey primarily explored a diversity of k13 mutations from imported cases imported from Africa between 2014 and 2019. Limited polymorphism of k13 was observed in our study, which indicated that k13 propeller domain of *P. falciparum* imported from African countries was still relatively conserved. However, the sample size of the current study was small, and more samples from different regions are required for confirmation. Notably, A676S substitution was verified in a 2106 isolate from Nigeria in the current survey. It was previously discovered in Ghana and southeast Asia [20–22], but it might be the first time in Nigeria. As for the association between A676S substitution and artemisinin resistance, therapeutic efficacy data from Ghana support that *P. falciparum* with this mutation is still susceptible to artemether–lumefantrine [20], however, there is still no evidence from in vitro tests. This warrants continuous surveillance to monitor this molecular marker, so as to evaluate its potential impact and association with drug resistance.

Although several publications are showing that short term culture of *P. vivax* is possible [23, 24], it is still difficult to reveal the underlying mechanisms of antimalarial drug resistance of *P. vivax*. However, evidence from several studies indicates that multiple molecular markers of *P. vivax* are involved in drug resistance, which are conferred from homologous genes in *P. falciparum*, such as *Pvdhps* and *Pvdhfr* [25, 26]. As k13 orthologue in *P. vivax*, k12 is a concern for researchers, although its role in artemisinin resistance is not demonstrated. There are several reports documenting the prevalence of mutant k12 in Cambodia, Thailand, Lao People’s Democratic Republic and the China–Myanmar border [13, 14, 27]. Generally speaking, previous studies have demonstrated that k12 mutation is rare, and all mutations are present at very low prevalence [13, 14, 27]. In our study, polymorphism of k12 was not observed in parasites from Africa or Asia countries, indicating that k12 is conserved in this region.

The current study was limited by a lack of clinical and in vitro data for the association between k13 mutation and artemisinin resistance. Further, there was a small sample size of *P. vivax*. and the contribution of several sites is limited to one or two isolates, which limited interpretation.

In conclusion, the current study demonstrated limited polymorphism in k13 gene of imported *P. falciparum* isolates from African countries, and our data presented, for the first time, A676S mutation of k13 in Nigeria. No evidence for the polymorphism of k12 in imported *P. vivax* samples from African and Asian countries was found. These data might be helpful for updating the evidence-based drug guidelines in study region.

## Supplementary Information

**Additional file 1: Table S1.** Primers and cycling conditions for *K13 and K12 *genotyping assay.

## Data Availability

The datasets analyzed in this study are available from the corresponding author on reasonable request. DNA sequences of k13 with the point mutations were deposited in the NCBI database under GenBank accession number MZ170790 to MZ170792.
