# Supplementary material for: Limited polymorphism in k13 gene of Plasmodium falciparum and k12 of Plasmodium vivax isolates imported from African and Asian countries between 2014 and 2019 in Hangzhou city, China
Source: BMC Infect Dis. 2021 Aug 21;21:853. doi: 10.1186/s12879-021-06579-6 (PMC8379771; doi:10.1186/s12879-021-06579-6)
Supplement: Supplementary file 1 — Additional file 1: Table S1. Primers and cycling conditions for K13 and K12 genotyping assay. [file 12879_2021_6579_MOESM1_ESM.docx]

Table S1 Primers and cycling conditions for *K13 and K12* genotyping assay

| Genes | Sequences (5’→3’) | Cycling conditions | Product size(bp) |
| --- | --- | --- | --- |
| *K13*(1st) | F: CGGAGTGACCAAATCTGGGA  R: GGGAATCTGGTGGTAACAGC | 95 ℃ 2 min, [95 ℃ 30 s,60 ℃ 90 s, 72 ℃ 90 s] ×30 cycles,72 ℃ 10 min | 2147 |
| *K13*(2nd) | F: GCCAAGCTGCCATTCATTTG  R: GCCTTGTTGAAAGAAGCAGA | 95 ℃ 2 min, [95 ℃ 30 s,60 ℃ 90 s, 72 ℃ 90 s] ×30 cycles,72 ℃ 10 min | 849 |
| *K12*-1 | F: ATGGAGGGCGAAAAAATAAAAT  R: CCTATCTAGAAATATCCTGCCTTG | 95 ℃ 5 min, [94 ℃ 30 s,58 ℃ 30 s, 72 ℃ 60 s] ×38 cycles,72 ℃ 10 min | 1152 |
| *K12*-2 | F: CGGAGACTATGATTGATATAAACGT  R: AATTAAAACGGAATGTCCAAATC | 95 ℃ 5 min, [94 ℃ 30 s,58 ℃ 30 s, 72 ℃ 60 s] ×38 cycles,72 ℃ 10 min | 1127 |
